# Morphometric Characterization and Preliminary Heritability Estimates of Body Measurements in the Polish Konik Populations

**DOI:** 10.3390/ani16081190

**Published:** 2026-04-14

**Authors:** Edyta Pasicka, Zbigniew Sobek, Heliodor Wierzbicki, Jolanta Różańska-Zawieja, Anna Nienartowicz-Zdrojewska

**Affiliations:** 1 Department of Biostructure and Animal Physiology, Faculty of Veterinary Medicine, Wrocław University of Environmental and Life Sciences, Kożuchowska 1/3, 51-631 Wroclaw, Poland; 2 Department of Genetics and Animal Breeding, Poznań University of Life Sciences, Wołyńska 33, 60-637 Poznan, Poland; zbigniew.sobek@up.poznan.pl (Z.S.); jolanta.rozanska-zawieja@up.poznan.pl (J.R.-Z.); anna.nienartowicz-zdrojewska@up.poznan.pl (A.N.-Z.); 3 Department of Genetics, Wrocław University of Environmental and Life Sciences, Kożuchowska 7, 51-631 Wroclaw, Poland; heliodor.wierzbicki@upwr.edu.pl

**Keywords:** correlations, discriminant analysis, heritability, horses, morphometrics, Poland, principal component analysis

## Abstract

The objective of this paper is to demonstrate the morphometric diversity of 172 Polish Koniks from the largest Polish breeding centers and to assess their genetic diversity by estimating the effective population size (*N_e_*) and heritabilities (*h*^2^) of the studied traits. The animals were bred under a stable housing system. Horses of both sexes aged 3 to 24 years were analyzed. Each horse was characterized with 40 metric traits. Multifactorial analyses (PCA, DA) and the REML method were applied to reveal the exterior differentiation and the development of distinct morphometric types. The findings established that the metric traits with high discriminant power include sections constituting segments of limbs. The elongation of segments, especially the pelvic limb—which serves a driving function—may be associated with deliberate breeding efforts aimed at improving the motor abilities of these horses. Estimates of heritability were high for eight traits, including sternal height (*h*^2^ = 0.81) and withers height (*h*^2^ = 0.75), while the effective population size was *N_e_* = 651. Although estimated with large standard errors due to the limited number of individuals studied, these results indicate the possibility of effectively conducting the genetic part of the conservation program for this breed. Koniks are a conservative breed, which creates an obligation for a comprehensive assessment of their current suitability for various forms of saddle and light draft use. Research using the methodology presented in this paper is worth repeating regularly to provide particularly valuable insights from a long-term perspective.

## Simple Summary

This paper examined 172 Polish Konik horses to understand their physical diversity and genetic background. The objective was to identify differences in their body shapes and assess how these traits are inherited, especially as these animals are increasingly used for riding and recreation. The findings established that distinct morphometric types exist within the population, which are characterized by specific limb segments and the high heritability of traits like height. Some traits, like longer limb segments, may be intentionally bred to improve the horses’ ability to move effectively. Although the genetic data were specific, the results suggest that selective breeding could help preserve and improve this breed. Overall, the findings are valuable for guiding breeding programs to maintain healthy, functional horses that meet modern use needs. Regularly repeating this type of research over many decades can help track changes in the breed and ensure its conservation and proper use in society.

## 1. Introduction

Polish Konik horses are a native Polish breed, originating from wild tarpans [1,2]. The first Polish Konik registry was started in 1955. On its basis, the first volume of the studbook for this breed was created in 1962, and since 1984, the registry has maintained closed studbooks. This entails running these operations exclusively in purity of breed, which is in accordance with the rules for stud book keeping [1,2,3,4,5,6,7,8,9,10]. In closed breeding programs, the inability to bring in a foreign bloodline also necessitates mating related individuals, which may lead to an increase in the inbreeding coefficient [10,11,12,13,14]. The numbers of the domestic population of this breed are small yet with a growing trend (according to data for 2021: approx. 2500 mares and 200 stallions, of which approx. 1700 mares and 180 stallions are active breeders) [15], which classifies this breed as endangered (in accordance with the FAO Programme for the Management of Farm Animal Genetic Resources based on the Convention on Biological Diversity) [16,17]. Distinctive for this breed are two different housing methods, stable housing and preserve housing, as well as low housing requirements, low quality feed conversion, healthiness, longevity, growth compensation, high draught force relative to body weight, and herd behavior strongly pronounced in reserve horses [4,6,8,10,18,19,20]. Partly due to these unique biological characteristics inherited from the tarpans, the Polish Konik breed has been covered since 1999 by a conservation breeding program [15]. The desired primitive horse-type conformation is mouse-dun or grullo color coat with a dark stripe across the back, without varieties, with rich mane and tail, and minimum biometric standards for adult horses: withers height: 130.0–140.0 cm, thorax circumference: 165.0 cm, metacarpal circumference: 16.5 cm (mares) and 17.5 cm (stallions) and since 2000. The genetic resource conservation program [5,7,10,17] ensures the genetic diversity of horses by preserving and increasing the population in size as well as preserving specific phenotypic and genetic characteristics. Polish Koniks are commonly recognized as a breed of uniform morphotype, although some authors draw attention to their progressing differentiation concerning type and conformation [8,19,20,21,22]. One of the main criteria during the selection of various animal species, including horses, is the evaluation of conformation, and the first external studies of Konik horses date back over a century to 1914 [23]. It seems well founded to conduct a detailed analysis based on morphometric measurements to assess morphological changes over this period as well as to estimate the genetic and phenotypic parameters that differentiate them. It is the opinion of the authors of this paper that its results will be a valuable contribution to the available literature in this field. The aims of the paper were to demonstrate the morphometric diversity of 172 Polish Koniks from the largest Polish breeding centers and to assess their genetic diversity by estimating the effective population size (*N_e_*) and heritabilities (*h*^2^) of the studied traits.

## 2. Materials and Methods

### 2.1. Animals

The study material consisted of stable bred Polish Konik horses from five leading Polish reserve breeding centers: Research Plant of Organic Farming and Preservative Animal Breeding PASc in Popielno (Warmińsko–Mazurskie Voivodship, Popielno, Poland), Roztocze National Park in Zwierzyniec (Lubelskie Voivodship, Zwierzyniec, Poland) (RPN), Stallions Herd Sieraków Wlkp. Ltd. (Wielkopolskie Voivodship, Sieraków, Poland), Horses Stud in Dobrzyniewo Ltd. (Wielkopolskie Voivodship, Dobrzyniewo, Poland) and Poznań Plants Breeding Ltd.–Branch in Kobylniki (Wielkopolskie Voivodship, Kobylniki, Poland).

### 2.2. Description of the Exterior Metric Traits

**Figure 1:**
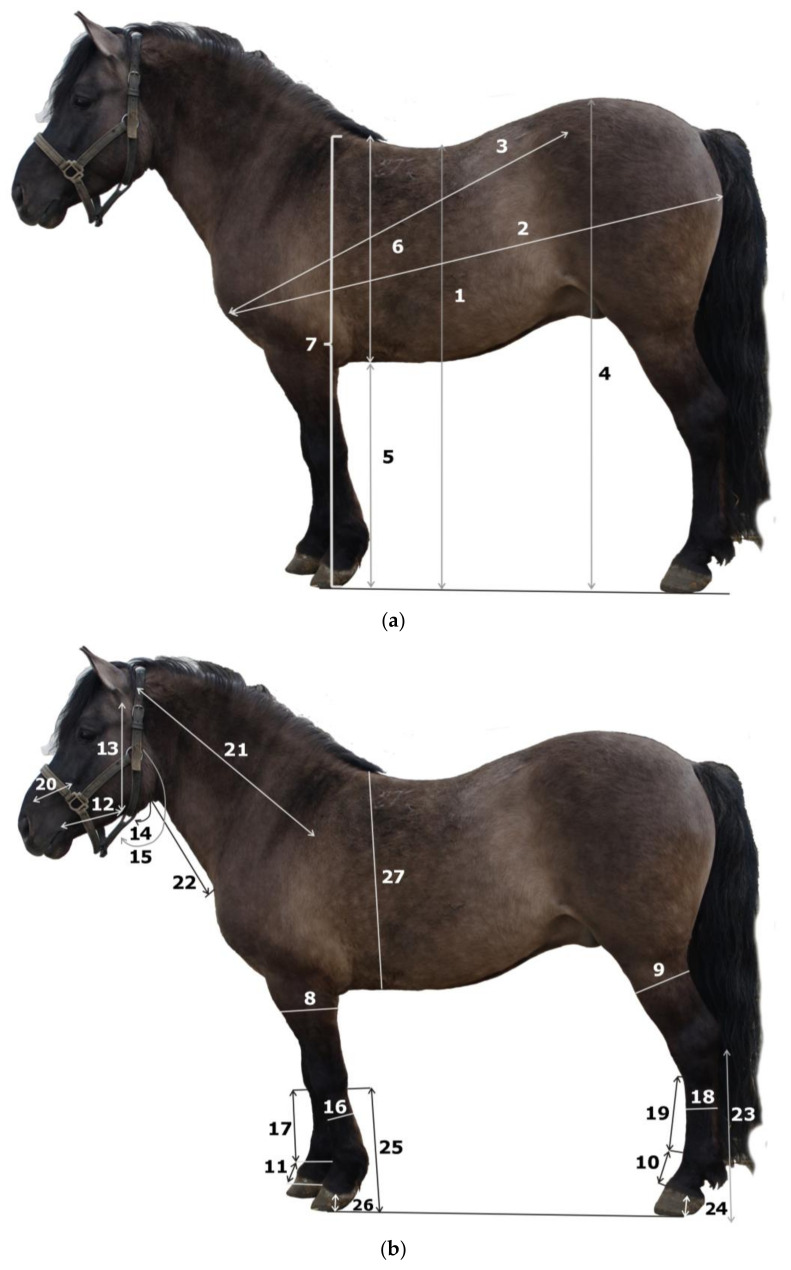

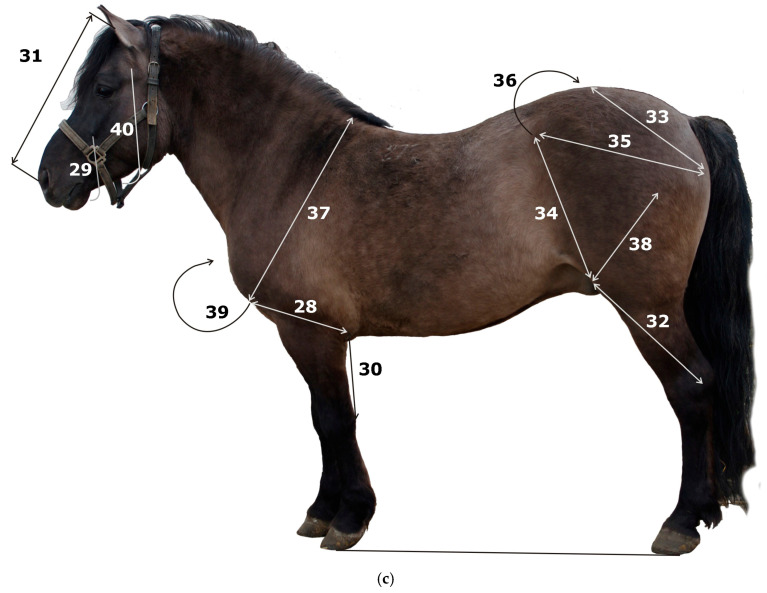
(**a**). Polish Konik horse, measurements of metric traits using zoometric stick (drawn by E. Pasicka). 1: **Back height (BH)** (distance between the highest point of back and the ground surface), 2: **Maximal oblique trunk length (MaxOTL)** (distance between the *tuberculum majus ossis humeri* and the *tuber ischiadicum*), 3: **Minimal oblique trunk length (MinOTL)** (distance between the *tuberculum majus ossis humeri* and the *tuber coxae*), 4: **Rump height (RH)** (distance between the highest point of the rump and the ground surface), 5: **Sternal height (StH)** (distance between the lowest point of the sternum and the ground surface), 6: **Thorax depth (ThD)** (distance between the highest point of the withers and the lowest ventral point of the sternum), 7: **Withers height (WH)** (distance between the highest point of the withers and the ground surface). (**b**). Polish Konik horse, measurements of metric traits using zoometric tape (drawn by E. Pasicka). 8: **Forearm circumference (FamC)** (on the half-length level of the forearm), 9: Leg circumference (LgC) (on the half-length level of the leg), 10: **Length of pelvic proximal and middle phalanx (LPvPMPh)** (distance between the fetlock joint and the *margo coronalis ungulae* of the pelvic limb), 11: **Length of thoracic proximal and middle phalanx (LThPMPh)** (distance between the fetlock joint and the *margo coronalis ungulae* of thoracic limb), 12: **Mandibular dimension I (MDI)** (distance between the vascular notch (*incisura vasorum facialium*) and the oral angle (*angulus oris*)), 13: **Mandibular dimension II (MDII)** (distance between the temporomandibular joint (*articulatio temporomandibularis*) and the vascular notch), 14: **Mandibular width I (MWI)** (distance between the most lateral points of right and left *ramus mandibulae*), 15: **Mandibular width II (MWII)** (distance between the most lateral points of right and left *ramus mandibulae* on the half height level of *ramus mandibulae*), 16: **Metacarpal circumference (McC)** (on the distal one-third length of the metacarpal bone), 17: **Metacarpal length (McL)** (distance between the *tuberositas ossis metacarpalis* and the fetlock joint (*articulatio metacarpophalangea*), 18: **Metatarsal circumference (MtC)** (on the distal one-third length of the metatarsal bone), 19: **Metatarsal length (MtL)** (distance between the *tuberositas ossis metatarsalis* and the fetlock joint (*articulatio metatarsophalangea*), 20: **Naso-facial dimension (NFcD)** (distance between the rostral end of *crista facialis* and the *incisura nasoincisiva*), 21: **Neck lateral length (NLL)** (distance between the auricular base (*basis auriculae*) and the half-length of *spina scapulae*), 22: **Neck ventral length (NVL)** (distance between the *basihyoideum* and the *apertura thoracis cranialis*), 23: **Pelvic autopodium length (PvAuL)** (distance between the *tuber calcanei* and the ground surface), 24: **Pelvic ungular height (PvUH)** (distance between the *margo coronalis ungulae* and the *margo solearis ungulae* of the pelvic limb), 25: **Thoracic autopodium length (ThAuL)** (distance between the *os carpi accessorium* and the ground surface), 26: **Thoracic ungular height (ThUH)** (distance between the *margo coronalis ungulae* and the *margo solearis ungulae* of the thoracic limb), 27: **Thorax circumference (ThC)** (along the line from the caudal angle of scapula (*angulus caudalis scapulae*) to the *tuber olecrani*). (**c**). Polish Konik horse, measurements of metric traits using zoometric caliper (drawn by E. Pasicka). 28: **Arm length (AmL)** (distance between the *tuberculum majus ossis humeri* and *tuber olecrani*), 29: **Facial width (FcW)** (distance between the end point of right and left *crista facialis*), 30: **Forearm length (FamL)** (distance between the *tuber olecrani* and the antebrachiocarpal joint (*articulatio antebrachiocarpea*)), 31: **Head length (HL)** (distance between the *apex nasi* and the *crista nuchae*), 32: **Leg length (LgL)** (distance between the *basis patellae* and the *malleolus lateralis*), 33: **Pelvic dimension I (PvDI)** (distance between the *processus spinosus vertebrae sacralis primae* and the *tuber ischiadicum*), 34: **Pelvic dimension II (PvDII)** (distance between the *tuber coxae* and the *basis patellae*), 35: **Pelvis length (PvL)** (distance between the *tuber coxae* and the *tuber ischiadicum*), 36: **Pelvis width (PvW)** (distance between the most lateral points of *tuber coxae*), 37: **Scapular length (with withers) (ScL)** (distance between *tuberculum majus ossis humeri* and the highest point of the withers (interscapular region)), 38: **Thigh length (TL)** (distance between the *trochanter major* and the *basis patellae*), 39: **Thorax width (ThW)** (distance between the right and left *tuberculum majus ossis humeri*), 40: **Zygomatic width (ZW)** (distance between the most lateral points of right and left *processus zygomaticus ossis frontalis*).

A total of 172 Polish Koniks were measured (46 males and 126 females) aged 3 to 24 years. To describe the exterior of Polish Koniks, for each horse, 40 measurements of metric traits were taken according to the methodologies presented on Figure 1a–c [20]. Anatomical terminology was used in accordance with the guidance of the Committee on Veterinary Gross Anatomical Nomenclature [24]. All measurements were performed by the first author of this paper on plain and stabilized ground. Side measurements were taken on the left side of the animal. The tools used were a Hauptner & Herberholz GmbH, Solingen, Germany zoometric stick, zoometric tape and zoometric caliper.

### 2.3. Statistical Analysis

Methods of multifactorial analyses have been applied for this investigation [25]. Principal Component Analysis (PCA) is a set of statistical procedures which, by transforming initial variables into new uncorrelated variables (components), produce a theoretical mathematical model in the form of systems of linear equations that describes the relational structure of the studied constituents.

Discriminant Analysis (DA) is a method used to determine whether groups differ based on the means of certain variables. The goal is to identify variables that can be used to predict group membership. In practice, multiple variables are often examined to assess which ones contribute most to distinguishing between the groups. It is a statistical procedure that allows studying differences between two or more groups, simultaneously analyzing several variables.

The statistics applied for this analysis are outlined below:

Wilks’ Lambda—indicates the statistical significance of discriminant power of a chosen model; its value fits between 1 (lack of discriminant power) and 0 (perfect discriminant power).

F-value—a greater value of this statistic indicates a greater discrimination of variables.

Partial Wilks’ Lambda—indicates the share of a chosen variable to discrimination of groups; the lower its value, the higher discriminant power of a given variable.

F-remove—the higher the value of this parameter for a variable, the higher its discriminant power. 

*p*-level—*p* is the critical probability level.

Numerical data were processed using the statistical–graphical software suite StatSoft Statistica 13 (StatSoft Inc., Tulsa, OK, USA).

### 2.4. Effective Population Size and Estimates of Heritability

The effective population size (*N_e_*) was estimated using the following formula:

$$N_{e}=\frac{4N_{m}N_{f}}{N_{m}+N_{f}}$$

where *N_m_* is the number of breeding males and *N_f_* is the number of breeding females. This formula was used to account for an uneven sex ratio in a breeding population.

To estimate the heritabilities (*h*^2^) of the studied traits, the single-trait animal model and the restricted maximum likelihood (REML) method was used. The DMU 6.4 package [26] and the average information (AI) algorithm were used in the estimation. The following mixed model was employed:

*Y_ijkl_* = *µ* + *age_i_* + *h_j_* + *s_k_* + *a_ijkl_* + *e_ijkl_*

where *Y_ijkl_* is the observation, *µ* is the population mean, *age_i_* is the fixed effect of age, *h_j_* is the fixed effect of stud, *s_k_* is the fixed effect of sex, *a_ijkl_* is the additive genetic effect of an animal, and *e_ijkl_* is the residual effect.

The complete pedigree information for all 172 individuals is provided in Table S1.

## 3. Results

Table 1 presents the mean values and standard deviations of the 40 metric traits analyzed in the entire Polish Konik population, which were divided into the five breeding centers from which the analyzed material originated. To examine the degree of morphometric diversity of the horses analyzed, multifactorial analyses were employed.

### 3.1. Multifactorial Analyses

**Figure 2:**
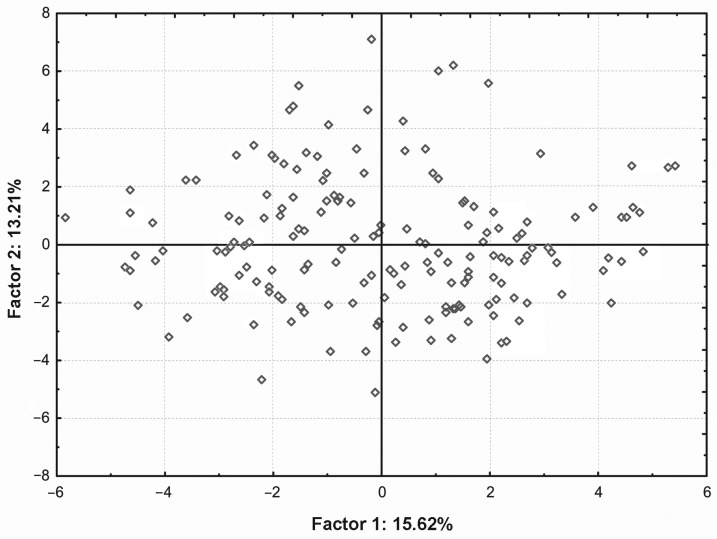
Factorial coordinates for 40 metric traits of Polish Koniks in range of Factor 1.

PCA: This method provided a description of the degree of morphometrical differentiation of the studied population of Polish Konik. On its basis, it was possible to examine the similarity of specimens with regard to all 40 metric traits jointly. From the array of 40 traits, the analysis set apart 12 new variables, which are the principal components. Combined, they presented 70.34% of the variability dependent on all variables. The factor 1 (first principal component) explained 15.62% of variability and the next (factor 2) explained 13.21%, as has been shown in a two-dimensional scatter graph (Figure 2), which allowed presenting the diverseness of the studied specimens with regard to the verified metric traits. Regarding factor 1, the investigated population of Polish Koniks was divided into groups, and it can be observed that the population is not homogeneous in terms of the described features.

DA: The objective of this analysis was to identify which of the 40 variables contributed to the greatest discrimination of the studied horses and furthermore to confirm the differentiation of the centers with regard to the studied metric traits. To accomplish this, the variable “center” was defined as grouping variable. Initially as a result of the conducted analysis, it was found—on the basis of the Wilks’ Lambda test statistics values, an approximate F statistic value and its corresponding *p* value—that the breeding center’s discrimination is highly significant (Wilks’ Lambda = 0.002, F (156.516) = 11.786, *p* < 0.000). A list of the variables that were identified as the most useful for discriminative analysis (*p* ≤ 0.05) is presented in Table 2. Eventually, a stepwise progressive analysis extracted 25 metric traits (MinOTL, MDII, ThC, TL, MWI, HL, WH, BH, LgC, PvDII, PvL, AmL, NFcD, NVL, McL, ThUH, NLL, MtC, StH, MtL, ThAuL, PvAuL, MWII, LThPMPh, and PvW) that were most strongly correlated with the morphotype of modern Polish Koniks (Table 3).

Next, we conducted a canonic analysis of discriminant function. The test outcome for discriminative functions established that the discriminative functions applied were statistically highly significant. This was confirmed by the high R canonic correlations of these functions, and the statistical significance of all discriminative functions is proven by the highly significant *p* values (Table 4).

Then, we specified the standardized coefficients of discriminative function (Table 5). With the help of these, one can compare the amounts and directions of each variables’ share in each canonic function; moreover, the percentage of variation explained by the functions applied in the model may be determined. In the research herein, 100% of the total discriminative power was explained by four functions. Hitherto, the results showed what share the variables had in the discrimination of centers.

To describe the nature of this discrimination, it is important to look at the means of the canonic variables (Table 6). These means enable the defining of groups that were most strongly differentiated by each canonic function. As arose from the data presented, the first discriminant function differentiates Popielno center foremost. Also, the second discriminant function deserves attention because it clearly distinguishes Sieraków and Roztocze National Park from the remaining centers. In addition, the third function distinguishes the Kobylniki and Dobrzyniewo centers from other centers, and the fourth points to the diversity of Roztocze National Park in relation to the other centers.

**Figure 3:**
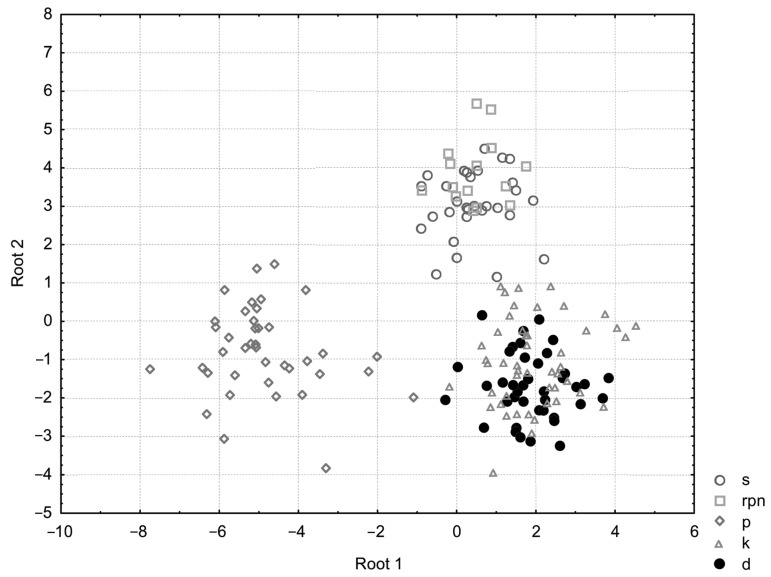
Diagram of canonical variable dispersion. Centers: Stallions Herd Sieraków Wlkp. Ltd. (s), Roztocze National Park in Zwierzyniec (rpn), Research Plant of Organic Farming and Preservative Animal Breeding PASc in Popielno (p), Poznań Plants Breeding Ltd.–Branch in Kobylniki (k), Horses Stud in Dobrzyniewo Ltd. (d).

In order to prove differentiation of the Koniks from five centers, canonic values were presented on a scatter plot (Figure 3). This plot confirms the outcomes obtained in the previous multifactorial analyses. A clear differentiation of the studied centers can be seen, and thus it shows discrepancies in the morphotypes of horses from the five largest Polish breeding centers. The analysis of standardized coefficients for canonical variables (Table 5) revealed that the first discriminant function (Root 1), which explains 46% of the total variation, was most strongly defined by three trunk measurements: minimal oblique trunk length (0.85), thorax circumference (−0.83), and back height (0.69). This indicates that these traits are the primary drivers of differentiation between the studied breeding centers. The second function (Root 2) was characterized by maximal oblique trunk length (0.56) and thoracic ungular height (0.46). Interestingly, the third function (Root 3) showed a very high correlation with metacarpal circumference (0.62) and thigh length (0.60). The cumulative proportion of the first three roots reached 93%, demonstrating an almost complete explanation of the morphometric diversity within the studied population.

### 3.2. Effective Population Size and Estimates of Heritability

The Polish Konik breed included in this paper originated from five leading Polish conservation breeding centers, making them unique and representative material. The conservation program for this breed assumes a limited number of representatives in the herd. The effective population size estimated for the studied population was *N_e_* = 651 (estimated for *N_m_* = 180 and *N_f_* = 1700).

Although the research material encompassed almost all animals kept at these centers (*n* = 172), their number should be considered very small for the estimation of heritabilities. Aware that a small number of individuals would affect the accuracy of the estimated genetic parameters, we decided to carry out their estimation in order to provide a latest, preliminary insight into the genetic determination of the traits studied. Heritabilities were estimated for 24 of the 40 measured metric traits that are important for maintaining the unique character of this breed. Therefore, despite their low accuracy, they provide valuable information on the effectiveness of the genetic part of the conservation program implemented for this breed as well as complement the first part of our research describing the morphology of the Polish Konik.

The estimated heritabilities (*h*^2^) turned out to be high for 8 out of the 24 examined conformation traits. In the group of eight high heritability traits (Table 7), the highest value of this parameter was estimated for StH (*h*^2^ = 0.81) and WH (*h*^2^ = 0.75), and the lowest was estimated for ZW (*h*^2^ = 0.47) and BH (*h*^2^ = 0.46). The remaining 16 of the 24 analyzed metric features proved to be moderate or low heritability traits (*h*^2^ = 0.02 to 0.38) (Table 7). The lowest value of heritability was estimated for MDI (*h*^2^ = 0.03) and for NFcD (*h*^2^ = 0.02) (Table 7). The standard errors for all estimated heritability coefficients were high (ranging from 0.25 for NFcD to 0.32 for StH), which is probably due to the very limited number of individuals used to estimate genetic parameters (this is due to the status of this breed—under a conservation program). However, as mentioned earlier, if these estimates are treated as a preliminary insight into the genetic determination of the traits that make the Polish Konik breed unique, they can provide valuable guidance on how to effectively implement a program for the conservation of this breed.

The estimation of heritability (*h*^2^) across anatomical regions provided a clear pattern of genetic determination. The highest genetic influence was observed in the trunk region particularly for sternal height (0.81) and withers height (0.75). In contrast, traits within the head and neck region showed extreme polarities: while the neck ventral length (0.62) was highly heritable, mandibular dimension II (0.03) and the naso-facial dimension (0.02) were almost entirely shaped by environmental factors. Furthermore, the stepwise discriminant analysis (Table 3) confirmed that the minimal oblique trunk length (Wilks’ Lambda 0.431) is the most significant trait for group membership prediction.

## 4. Discussion

Based on metric traits, the size tendencies in the breeding of different breeds of horses can be described, and exterior changes that took place during many years of a given breed’s existence can be assessed as well [20,27,28,29,30,31,32,33,34].

Wrześniowski [18] was conducting research on the conformation and breeding of Polish Konik horses of the Vilnius region, and he concluded that Koniks in the 1930s showed greater alignment with respect to certain dimensions. Mares were more aligned compared to geldings with respect to the trunk length, head length, neck length, thorax depth, width of the hips and cannon circumference. Geldings, however, showed greater uniformity in the withers height, width of the forehead and mouth, and thorax width as compared to mares from the 1930s.

According to the findings of Kownacki [19], the proportions of Polish Koniks build, which were measured in the years 1914 to 1979 (by various authors and in different regions of Poland), underwent quite significant changes, while their growth, strongly determined genetically, in the analyzed period of time was slightly increased only at the end of the 1980s. Kownacki [19], on the basis of means of biometric measurements, concluded that Polish Koniks increased their dimensions first of all in thorax circumference, metacarpal circumference, and in the period analyzed by him that had a beneficial impact on their utilitarian features as draught horses in agriculture.

Studies conducted by Pietrzak et al. [21], which aimed to describe the characteristics of Polish Koniks from the main breeding centers, showed that by the late 1980s, most of the horses in the breed type were kept in reserves. In our own research, horses from reserves were not measured; however, at the Popielno facility, the barn breeding of Polish Koniks is adjacent to reserve breeding, and horses from the reserve are introduced into barn breeding (reverse introduction is not permitted in Polish Konik breeding). This may explain why horses from Popielno differ in exterior from those at the other four studied centers. Also, Pietrzak and Wojciechowski [35] found that at the end of the 1990s, Polish Koniks that were kept in the main centers of stable, out-of-stable and reserve breeding differed with regard to some metric traits. Stable stallions stood out with rump height higher than out-of-stable stallions. Concerning the measurements of the head, thorax and rump, stable mares from Popielno statistically significantly outmatched mares from other facilities. Stable breed mares and stallions differed highly significantly from the out-of-stable specimens with regard to oblique trunk length. Koniks kept in Zwierzyniec reserve presented lower indices than specimens from other facilities where this breed is bred.

Withers height (WH) is one of the more important phenotypic parameters and plays a significant role in allocating animals, including horses, to a specific utilitarian type [30,31,32,33,34]. In this paper, the heritability coefficient for this trait (WH) was high (*h*^2^ = 0.75). Similar results (*h*^2^ = 0.65) were reported by Wolc and Balińska [11], and it was higher than the value of 0.46 obtained by Kaproń et al. [36]. Higher values of this coefficient for SH were found in the studies of Kaproń and Pluta [37] for Hucul horses (*h*^2^ = 0.83), Henk et al. [38] for Shetland ponies (*h*^2^ = 0.89), and Hintz et al. [39] for Thoroughbreds (*h*^2^ = 0.88).

WH, being a trait strongly determined by genetics in the Polish Konik population, has not undergone significant growth. Since the first documented research carried out by Grabowski and Schuch [23], it remains on a steady level above 130.0 cm. When comparing the mean values of metric traits from our own research to the analogous metric traits from the study by Grabowski and Schuch [23] (WH = 133.8 cm; BH = 127.3 cm; RH = 133.0 cm; MaxOTL = 140.6 cm; ThC = 161.3 cm), an increasing trend is clearly visible. This increasing trend in the mean values of these metric traits is also confirmed by the study by Komosa and Frąckowiak [22] (WH = 135.3 cm; RH = 137.3 cm; MaxOTL = 143.3 cm; McC = 17.9 cm; ThW = 36.8 cm; PvW = 46.6 cm), and the values for these traits are consistent with the values obtained in this paper. Nowadays, the withers height mean value for the investigated horses, similarly to the mean values for thorax circumference and metacarpal circumference—the other two standard measurements—falls within the current biometric template for this breed [9,15,17]. Our findings regarding the core biometric parameters (WH, ThC, McC) are in full alignment with the long-term trends described in previous studies [19,21,22] This confirms that the Polish Konik population maintains a steady, gradual increase in overall body size, which is a phenomenon typical for primitive breeds when housing conditions are optimized. The fact that current means remain within the breed standard reflects the effectiveness of existing phenotypic monitoring within the conservation program. Regarding the characteristic ‘heavy head’ that is often attributed to this breed, our findings provide a nuanced perspective based on the observed gradient of heritability. While the breed standard and literature frequently emphasize a massive head as a hallmark of the primitive morphotype—a view partly supported by the high heritability of structural dimensions like MWII and ZW and the moderate heritability of HL—our estimates for specific mandibular and naso-facial dimensions (MDI and NFcD) were remarkably low (*h*^2^ ≤ 0.03). This suggests that while the overall skeletal frame of the head is genetically anchored, certain proportions and the perceived ‘heaviness’ are not fixed genetic traits. Instead, these specific dimensions appear highly plastic and strongly influenced by environmental and developmental factors rather than conscious breeding selection. Consequently, these particular elements of the head morphotype may act more as indicators of rearing conditions or individual ontogeny than stable markers of breed purity.

The high discriminant power of limb segments, such as thigh length and metatarsal length, supports the hypothesis that modern breeding selection—even in conservation programs—is subtly shifting toward horses with improved movement dynamics.

A key novelty of this paper, distinguishing it from earlier descriptive reports, is the identification of specific limb segments (particularly thigh length and metatarsal length) as the primary drivers of internal population differentiation. While previous literature qualitatively suggested a shift toward ‘better movement,’ our multifactorial analysis provides quantitative evidence that these traits are the actual ‘markers’ of the transition from a primitive to a more refined saddle-type morphotype. Furthermore, by linking 40 metric traits with preliminary heritability estimates, we have identified which characteristics are ‘resistant’ to selection (low *h*^2^ for head dimensions) versus those that respond most rapidly to current breeding fashions.

Recent studies on the conformation of Polish Koniks [20,22,40] indicate the formation of morphotypes within the population of the analyzed breed, which is indicated by the high differentiation of Polish Koniks with numerous metric traits. Nowicka-Posłuszna and Białkowski [41] believe that the progressive increase in the withers height of this breed is a result of the selection of Koniks toward riding.

In view of the growing interest of saddle use for the breed discussed, especially in recreation, more emphasis is placed on the assessment of motion of these horses, which is reflected in courage tests and exhibitions of stable bred Koniks [20]. According to the observations of Komosa [42], breeders sometimes choose individuals with better gaits dynamics, as evidenced by higher marks on exhibitions awarded to Koniks whose exterior resembles a noble horse. A preference for traits in Koniks that are typical for sport horses, such as gait quality or stride length, diverges from the reserve breeding rules for this breed.

According to the postulates of the breeding program of Polish Konik horses genetic resource conservation from 2009, traits related with recreational use should be perfected, paying attention to motion; however, it must not be at the cost of a major change in the type of Polish Konik, which should remain in its type approximate to primal and Tarpan-like [7].

A solution for those breeders who want to perfect Polish Koniks with regard to increasingly better motion predispositions is a quite new direction—the production of Konik-like ponies, where the ponies inherit traits of vitality after Koniks, and the second parent introduces sport traits [34,42].

In this paper, it was proven that based on analysis of discriminant function—a stepwise progressive method, the metric traits with high discriminative power are, among others, sections which constitute segments of limbs. Thus, it was statistically confirmed that among others, elements of pelvic limb build (thigh length, pelvic dimension II, metatarsal length and pelvic autopodium length) and thoracic extremity (arm length, metacarpal length, thoracic autopodium length and the length of thoracic proximal and middle phalanx) influence statistically significantly the exterior diversity of the horses analyzed as well as the creation of morphotypes within the studied population of animals.

The results of this paper confirm the earlier findings by Komosa and Frąckowiak [22], which showed that thigh length is one of the traits with significant discriminative power for describing the exterior of the horse breed in question. According to these authors, the elongation of this section of the pelvic extremity is also associated with improved movement dynamics.

Interesting observations were made by Kobryń [43] studying the morphometric features of horses dated back from Neolith to Middle Ages. These observations indicate that in the aforementioned period, the middle phalanx in thoracic extremity underwent the biggest changes, which was followed by the third metacarpal bone and radius. Kobryń [43] concluded that there is a fundamental relationship between the regularity for this limb, i.e., the farther a given bone is from the axial skeleton, the greater the changes that affect that bone. Kobryń [43] did not observe an analogous relation in case of pelvic limb, where he established that the middle phalanx underwent the biggest transformations, which was followed by the tibia and third metatarsal bone.

The difference concerning changes, which subsequent segments in both limbs are subject to, may be due to their different biomechanical function. The pelvic limb has primarily a driving function, and therefore it is most probably subject to multidirectional developmental changes; it is also more dependent on environmental morpho-creative factors [42,43]. Komosa and Frąckowiak [22] indicate such morpho-creative factors can include the selection of Polish Koniks toward saddle features.

To summarize this section, which is devoted mainly to discussing the morphology of the Polish Konik breed, it is worth mentioning the genetic parameters of the traits studied. The full picture of the breed in the conservation program consists not only of its phenotype but also of the gene pool that determines it. The knowledge of genetic parameters is crucial for endangered or rare breed conservation programs, as it allows breeders to develop effective breeding strategies that maximize genetic diversity, minimize inbreeding and improve adaptability to environmental changes. The effective population size (*Ne*) indicates that the census population of 1880 individuals may experience genetic drift and inbreeding at a rate comparable to that of an idealized population of only 651 individuals. Such a markedly reduced effective population size indicates the population’s heightened susceptibility to inbreeding depression and diminished genetic diversity. The genetic parameters provide a roadmap for conservation breeding, enabling data-driven decisions important for the long-term survival and viability of breeds and the preservation of the gene pool of endangered or rare breeds. Therefore, despite their low accuracy, the reported heritabilities may constitute additional valuable information for conservation decision making regarding the Polish Konik breed.

From a practical management perspective, these results offer concrete decision-making tools for breeders. First, the high heritability of withers height (*h*^2^ = 0.75) and sternal height (*h*^2^ = 0.81) suggests that these traits can be very effectively controlled through mate selection to prevent the breed from becoming too ‘leggy.’ Second, the identified discrepancy between the census population and the effective population size (*Ne* = 651) serves as a critical warning. It indicates the need for a more active exchange of breeding material between the five main centers to mitigate inbreeding depression. Breeders are thus provided with a clear guideline: selecting for ‘enhanced movement’ or ‘elongated lines’—traits with high discriminant power—will lead to a rapid loss of the unique Tarpan-like phenotype, which should be countered by strict adherence to minimum biometric standards during studbook entry.

## 5. Conclusions

This paper demonstrates that the Polish Konik population currently exhibits significant morphological diversity with distinct morphotypes emerging across different breeding centers. This differentiation highlights a shift toward a more functional, recreational conformation in some groups, which may pose a risk to the preservation of the breed’s original primitive characteristics. The genetic analysis reveals a clear distinction between highly heritable traits, such as body height, and those more susceptible to environmental influences, like head dimensions. This underlines the potential for effective selective breeding in maintaining the breed’s standard. However, the identified discrepancy between the effective population size and the actual number of horses serves as a critical warning regarding the population’s vulnerability to inbreeding and genetic drift. To ensure the long-term survival of the Polish Konik in its ancestral form, conservation management should prioritize the maintenance of the “Tarpan-type” phenotype over recreational utility. Key recommendations include a strict adherence to closed studbook rules, collaborative mating strategies across centers to maximize genetic diversity, and regular morphometric monitoring to assess the effectiveness of the ongoing conservation program.

## Figures and Tables

**Table 1:** Statistical characteristics (mean ± SD) of 40 metric traits of Polish Koniks (cm).

Anatomical Region/ Trait (Abbr.)	Sieraków (*n* = 30)	RPN (*n* = 15)	Popielno (*n* = 39)	Kobylniki (*n* = 48)	Dobrzyniewo (*n* = 40)	Total ∑
I. Head and Neck						
Head length (HL)	48.9 ± 2.56	45.2 ± 1.78	46.7 ± 3.39	47.6 ± 2.57	50.6 ± 1.91	47.8 ± 1.85
Zygomatic width (ZW)	21.1 ± 0.57	20.7 ± 0.96	21.0 ± 0.90	20.5 ± 0.81	20.6 ± 0.62	20.8 ± 0.23
Facial width (FcW)	13.7 ± 0.50	13.6 ± 0.37	13.9 ± 0.49	14.1 ± 0.82	13.5 ± 0.53	13.8 ± 0.22
Naso-facial dimension (NFcD)	11.8 ± 0.63	12.2 ± 0.88	11.8 ± 0.72	13.2 ± 0.98	12.8 ± 0.76	12.4 ± 0.56
Mandibular dimension I (MDI)	18.2 ± 1.22	17.8 ± 0.98	17.4 ± 0.95	18.4 ± 1.24	18.5 ± 1.33	18.1 ± 0.41
Mandibular dimension II (MDII)	27.0 ± 2.07	27.2 ± 1.08	28.3 ± 1.93	31.0 ± 1.29	31.0 ± 1.33	28.9 ± 1.77
Mandibular width I (MWI)	14.7 ± 0.84	14.2 ± 1.24	13.9 ± 1.05	16.5 ± 1.31	15.3 ± 0.85	14.9 ± 0.92
Mandibular width II (MWII)	38.0 ± 2.68	35.3 ± 3.11	35.8 ± 2.78	36.1 ± 2.35	35.6 ± 2.24	36.2 ± 0.96
Neck lateral length (NLL)	70.3 ± 3.35	66.6 ± 3.83	69.2 ± 2.87	71.4 ± 3.70	73.1 ± 3.24	70.1 ± 2.18
Neck ventral length (NVL)	41.1 ± 3.99	43.6 ± 4.24	40.6 ± 4.21	39.3 ± 3.28	42.6 ± 1.82	41.4 ± 1.51
II. Trunk						
Withers height (WH)	135.8 ± 1.97	136.8 ± 3.83	136.8 ± 3.88	133.4 ± 2.96	134.8 ± 2.88	135.5 ± 1.29
Rump height (RH)	138.3 ± 2.02	139.6 ± 2.75	138.6 ± 3.31	138.1 ± 2.87	137.7 ± 3.06	138.5 ± 0.64
Back height (BH)	130.5 ± 2.37	132.5 ± 3.18	130.1 ± 4.05	130.7 ± 2.89	129.5 ± 3.41	130.7 ± 1.01
Sternal height (StH)	71.6 ± 1.86	71.1 ± 2.72	71.5 ± 3.34	68.0 ± 2.71	67.5 ± 2.83	69.9 ± 1.80
Thorax depth (ThD)	64.3 ± 2.03	65.6 ± 1.99	65.4 ± 3.41	65.4 ± 2.92	67.3 ± 2.77	65.6 ± 0.97
Thorax width (ThW)	35.2 ± 2.29	36.6 ± 2.27	34.3 ± 4.08	35.8 ± 1.86	36.8 ± 2.27	35.7 ± 0.92
Thorax circumference (ThC)	166.4 ± 4.96	167.7 ± 5.39	182.0 ± 8.01	173.5 ± 6.29	171.1 ± 6.54	172.1 ± 5.53
Min. oblique trunk length (MinOTL)	113.6 ± 3.62	111.9 ± 2.71	102.5 ± 6.91	114.8 ± 3.57	115.3 ± 3.26	111.6 ± 4.71
Max. oblique trunk length (MaxOTL)	143.1 ± 4.01	141.6 ± 4.47	135.2 ± 6.28	142.0 ± 4.70	143.5 ± 4.10	141.1 ± 3.02
III. Pelvis						
Pelvis width (PvW)	47.2 ± 2.08	47.9 ± 1.33	46.6 ± 2.57	47.4 ± 2.32	49.4 ± 1.80	47.7 ± 0.95
Pelvis length (PvL)	44.7 ± 1.99	43.5 ± 2.82	41.9 ± 3.62	41.1 ± 2.41	43.5 ± 1.85	42.9 ± 1.28
Pelvic dimension I (PvDI)	44.2 ± 1.77	43.8 ± 2.23	42.7 ± 2.78	41.3 ± 2.12	43.5 ± 1.78	43.1 ± 1.03
Pelvic dimension II (PvDII)	41.9 ± 3.16	42.2 ± 1.93	42.4 ± 3.33	40.1 ± 2.29	36.7 ± 2.17	40.7 ± 2.14
IV. Forelimbs						
Scapular length (ScL)	56.0 ± 2.04	56.3 ± 2.14	58.0 ± 1.92	56.9 ± 1.73	57.8 ± 1.40	57.0 ± 0.79
Arm length (AmL)	33.2 ± 1.54	31.3 ± 1.42	33.9 ± 1.82	32.5 ± 2.20	31.8 ± 1.79	32.5 ± 0.94
Forearm length (FamL)	37.6 ± 2.12	37.4 ± 1.46	36.2 ± 2.67	36.3 ± 3.02	36.0 ± 2.62	36.7 ± 0.66
Forearm circumference (FamC)	42.2 ± 2.98	45.6 ± 4.20	42.5 ± 2.57	41.3 ± 3.10	43.5 ± 3.67	43.0 ± 1.47
Metacarpal circumference (McC)	18.0 ± 0.64	18.5 ± 1.19	18.3 ± 0.89	19.0 ± 0.93	18.4 ± 0.70	18.4 ± 0.33
Metacarpal length (McL)	22.7 ± 2.07	21.5 ± 0.57	23.2 ± 2.26	23.7 ± 2.30	24.2 ± 1.33	23.1 ± 0.93
Thoracic autopodium length (ThAuL)	41.0 ± 1.02	42.0 ± 2.58	42.6 ± 1.67	40.8 ± 2.16	42.5 ± 2.55	41.8 ± 0.75
L. of thor. prox. & mid. phalanx (LThPMPh)	10.6 ± 0.47	10.7 ± 0.46	11.3 ± 0.86	11.1 ± 0.69	10.9 ± 0.34	10.9 ± 0.26
Thoracic ungular height (ThUH)	5.3 ± 0.56	5.4 ± 0.80	5.5 ± 0.53	4.7 ± 0.53	4.7 ± 0.39	5.12 ± 0.35
V. Hindlimbs						
Thigh length (TL)	34.6 ± 2.37	37.3 ± 2.18	36.4 ± 3.12	35.9 ± 1.71	32.1 ± 1.53	35.3 ± 1.81
Leg length (LgL)	46.0 ± 3.36	44.3 ± 2.51	45.1 ± 4.66	46.0 ± 3.78	50.3 ± 3.51	46.3 ± 2.08
Leg circumference (LgC)	41.6 ± 1.81	42.4 ± 2.44	42.9 ± 3.55	42.9 ± 2.87	45.9 ± 2.81	43.1 ± 1.46
Metatarsal circumference (MtC)	20.6 ± 2.25	20.4 ± 1.50	20.6 ± 0.85	21.4 ± 0.91	20.8 ± 0.66	20.8 ± 0.34
Metatarsal length (MtL)	27.5 ± 2.48	26.5 ± 1.89	29.7 ± 2.67	30.2 ± 2.82	31.4 ± 1.37	29.1 ± 1.80
Pelvic autopodium length (PvAuL)	49.9 ± 1.77	48.9 ± 2.69	52.4 ± 2.77	50.2 ± 2.93	50.4 ± 2.14	50.4 ± 1.14
L. of pelv. prox. and mid. phalanx (LPvPMPh)	10.8 ± 0.55	11.1 ± 0.43	11.5 ± 1.06	11.4 ± 0.96	11.1 ± 0.46	11.2 ± 0.25
Pelvic ungular height (PvUH)	5.1 ± 0.51	5.2 ± 0.49	5.4 ± 0.48	4.9 ± 0.53	5.0 ± 0.42	5.1 ± 0.17

*n*: number of horses; SD: standard deviation; ∑: overall mean for five centers.

**Table 2:** Discriminant Function Analysis (grouping variable: center (5 groups) for morphometric traits of Polish Konik horses.

Trait (Abbr.)	Full Trait Name	Anatomical Region	Wilks’ Lambda	Partial Wilks’ Lambda	F-Remove	*p*-Level
ThC	Thorax circumference	II. Trunk	0.003	0.681	15.104	0.000
TL	Thigh length	V. Hindlimbs	0.003	0.776	9.311	0.000
MinOTL	Min. oblique trunk length	II. Trunk	0.003	0.794	8.368	0.000
MDII	Mandibular dimension II	I. Head and Neck	0.003	0.809	7.593	0.000
HL	Head length	I. Head and Neck	0.003	0.837	6.286	0.000
BH	Back height	II. Trunk	0.003	0.847	5.815	0.000
MWI	Mandibular width I	I. Head and Neck	0.003	0.856	5.444	0.000
WH	Withers height	II. Trunk	0.003	0.865	5.036	0.001
NVL	Neck ventral length	I. Head and Neck	0.003	0.871	4.768	0.001
NFcD	Naso-facial dimension	I. Head and Neck	0.003	0.871	4.760	0.001
McC	Metacarpal circumference	IV. Forelimbs	0.003	0.881	4.371	0.002
MtL	Metatarsal length	V. Hindlimbs	0.003	0.896	3.756	0.006
AmL	Arm length	IV. Forelimbs	0.003	0.896	3.724	0.007
ScL	Scapular length	IV. Forelimbs	0.003	0.901	3.529	0.009
ThUH	Thoracic ungular height	IV. Forelimbs	0.003	0.902	3.524	0.009
NLL	Neck lateral length	I. Head and Neck	0.003	0.920	2.806	0.028
ThAuL	Thoracic autopodium length	IV. Forelimbs	0.003	0.921	2.762	0.030
MaxOTL	Max. oblique trunk length	II. Trunk	0.003	0.923	2.676	0.035
McL	Metacarpal length	IV. Forelimbs	0.003	0.926	2.571	0.041
MWII	Mandibular width II	I. Head and Neck	0.003	0.929	2.466	0.048
FamL	Forearm length	IV. Forelimbs	0.003	0.932	2.356	0.057
ThW	Thorax width	II. Trunk	0.003	0.938	2.119	0.082
StH	Sternal height	II. Trunk	0.003	0.941	2.031	0.094
PvW	Pelvis width	III. Pelvis	0.003	0.941	2.016	0.096
FamC	Forearm circumference	IV. Forelimbs	0.003	0.941	2.007	0.097
PvDII	Pelvic dimension II	III. Pelvis	0.003	0.944	1.908	0.113
PvAuL	Pelvic autopodium length	III. Pelvis	0.003	0.947	1.789	0.135
FcW	Facial width	I. Head and Neck	0.003	0.948	1.764	0.140
LgC	Leg circumference	V. Hindlimbs	0.003	0.948	1.761	0.141
MtC	Metatarsal circumference	V. Hindlimbs	0.002	0.954	1.539	0.195
PvL	Pelvis length	III. Pelvis	0.002	0.959	1.389	0.241
LThPMPh	Length of thoracic phalanges	IV. Forelimbs	0.002	0.962	1.262	0.288
LPvPMPh	Length of pelvic phalanges	V. Hindlimbs	0.002	0.975	0.828	0.510
ZW	Zygomatic width	I. Head and Neck	0.002	0.976	0.778	0.542
RH	Rump height	II. Trunk	0.002	0.977	0.769	0.548
PvDI	Pelvic dimension I	III. Pelvis	0.002	0.981	0.633	0.640
PvUH	Pelvic ungular height	III. Pelvis	0.002	0.985	0.481	0.749
LgL	Leg length	V. Hindlimbs	0.002	0.987	0.421	0.793
MDI	Mandibular dimension I	I. Head and Neck	0.002	0.990	0.332	0.856

Significant statistics *p* ≤ 0.05.

**Table 3:** Progressive stepwise analysis summary for morphometric traits of Polish Konik horses.

Trait (Abbr.)	Full Trait Name	Anatomical Region	Wilks’ Lambda	F-Value	*p*-Level
MinOTL	Min. oblique trunk length	II. Trunk	0.431	55.148	0.000
MDII	Mandibular dimension II	I. Head and Neck	0.212	48.694	0.000
ThC	Thorax circumference	II. Trunk	0.111	47.156	0.000
TL	Thigh length	V. Hindlimbs	0.073	42.600	0.000
MWI	Mandibular width I	I. Head and Neck	0.053	38.494	0.000
HL	Head length	I. Head and Neck	0.038	36.437	0.000
WH	Withers height	II. Trunk	0.030	34.070	0.000
BH	Back height	II. Trunk	0.025	31.838	0.000
LgC	Leg circumference	V. Hindlimbs	0.021	29.925	0.000
PvDII	Pelvic dimension II	III. Pelvis	0.018	28.170	0.000
PvL	Pelvis length	III. Pelvis	0.015	27.014	0.000
AmL	Arm length	IV. Forelimbs	0.014	25.747	0.000
NFcD	Naso-facial dimension	I. Head and Neck	0.012	24.629	0.001
NVL	Neck ventral length	I. Head and Neck	0.011	23.634	0.002
McL	Metacarpal length	IV. Forelimbs	0.010	22.804	0.002
ThUH	Thoracic ungular height	IV. Forelimbs	0.009	22.133	0.001
NLL	Neck lateral length	I. Head and Neck	0.008	21.556	0.001
McC	Metacarpal circumference	IV. Forelimbs	0.007	21.103	0.001
StH	Sternal height	II. Trunk	0.006	20.439	0.012
MtL	Metatarsal length	V. Hindlimbs	0.006	19.803	0.019
ThAuL	Thoracic autopodium length	IV. Forelimbs	0.005	19.321	0.008
PvAuL	Pelvic autopodium length	III. Pelvis	0.005	18.783	0.025
MWII	Mandibular width II	I. Head and Neck	0.005	18.263	0.036
LThPMPh	Length of thoracic phalanges	IV. Forelimbs	0.004	17.772	0.044
PvW	Pelvic width	III. Pelvis	0.003	15.750	0.037

Significant statistics *p* ≤ 0.05.

**Table 4:** Chi-square tests of subsequent roots.

Roots	Eigenvalue	Canonical R	Wilks’ Lambda	Chi-Square	Df	*p*-Level
0	7.462	0.939	0.002	900.678	156	0.000
1	4.269	0.900	0.020	582.475	114	0.000
2	3.490	0.882	0.106	334.865	74	0.000
3	1.108	0.725	0.474	111.095	36	0.000

Significant statistics *p* ≤ 0.05; Df—degrees of freedom.

**Table 5:** Standardized coefficients of discriminant function for morphometric traits of Polish Konik horses.

Trait (Abbr.)	Full Trait Name	Anatomical Region	Root 1	Root 2	Root 3	Root 4
NLL	Neck lateral length	I. Head and Neck	0.10	−0.07	−0.14	0.44
NVL	Neck ventral length	I. Head and Neck	0.29	0.15	−0.04	−0.47
WH	Withers height	II. Trunk	−0.57	0.34	−0.55	−0.06
StH	Sternal height	II. Trunk	−0.36	−0.07	−0.05	0.26
RH	Rump height	II. Trunk	0.01	−0.25	0.15	0.08
ThC	Thorax circumference	II. Trunk	−0.83	−0.39	0.20	0.33
MinOTL	Min. oblique trunk length	II. Trunk	0.85	0.22	0.07	0.12
BH	Back height	II. Trunk	0.69	0.38	0.56	−0.26
MaxOTL	Max. oblique trunk length	II. Trunk	−0.21	0.56	−0.01	0.00
ThW	Thorax width	II. Trunk	0.27	−0.04	−0.27	−0.03
PvW	Pelvis width	III. Pelvis	−0.01	−0.05	−0.14	−0.40
PvL	Pelvis length	III. Pelvis	0.32	0.06	−0.18	0.38
PvDI	Pelvic dimension I	III. Pelvis	−0.14	0.14	−0.22	−0.19
PvDII	Pelvic dimension II	III. Pelvis	−0.21	0.16	0.23	0.00
ScL	Scapular length	IV. Forelimbs	0.08	−0.39	−0.21	−0.26
AmL	Arm length	IV. Forelimbs	−0.05	0.04	−0.06	0.52
FamL	Forearm length	IV. Forelimbs	−0.15	0.03	0.03	0.43
FamC	Forearm circumference	IV. Forelimbs	0.03	0.25	−0.01	−0.28
ThAuL	Thoracic autopodium length	IV. Forelimbs	−0.21	−0.25	−0.25	−0.20
McC	Metacarpal circumference	IV. Forelimbs	0.11	−0.02	0.62	−0.21
McL	Metacarpal length	IV. Forelimbs	−0.35	0.04	−0.01	0.15
LThPMPh	Length of thoracic phalanges	IV. Forelimbs	0.10	−0.22	0.10	−0.01
ThUH	Thoracic ungular height	IV. Forelimbs	0.02	0.46	−0.14	−0.08
TL	Thigh length	V. Hindlimbs	−0.03	0.29	0.60	−0.29
LgL	Leg length	V. Hindlimbs	0.13	−0.03	−0.06	−0.06
LgC	Leg circumference	V. Hindlimbs	−0.24	−0.05	−0.20	−0.15
PvAuL	Pelvic autopodium length	III. Pelvis	−0.18	−0.14	−0.13	0.15
MtC	Metatarsal circumference	V. Hindlimbs	0.21	−0.18	−0.10	0.08
MtL	Metatarsal length	V. Hindlimbs	0.03	−0.45	−0.18	−0.09
LPvPMPh	Length of pelvic phalanges	V. Hindlimbs	−0.12	0.00	0.18	−0.10
PvUH	Pelvic ungular height	III. Pelvis	−0.09	−0.03	0.15	−0.06
Eigenvalue			7.46	4.27	3.49	1.11
Cumulated proportion			0.46	0.72	0.93	1.00

**Table 6:** Mean canonical variables.

Center	Root 1	Root 2	Root 3	Root 4
Sieraków	0.444	3.084	−1.210	1.459
Roztocze National Park	0.446	3.923	1.122	−2.614
Popielno	−4.840	−0.841	0.145	−0.019
Dobrzyniewo	1.958	−1.051	2.370	0.375
Kobylniki	1.869	−1.704	−2.498	−0.545

**Table 7:** Heritability (*h*^2^) of conformation traits in Polish Koniks across anatomical regions.

Anatomical Region	High (*h*^2^ > 0.40)	Moderate (*h*^2^ = 0.20–0.40)	Low (*h*^2^ < 0.20)
I. Head and Neck	NVL (0.62)—Neck ventral length MWII (0.48)—Mandibular width II ZW (0.47)—Zygomatic width	HL (0.36)—Head length	MDI (0.03)—Mandibular dimension I NFcD (0.02)—Naso-facial dimension
II. Trunk	StH (0.81)—Sternal height WH (0.75)—Withers height ThD (0.52)—Thorax depth BH (0.46)—Back height	RH (0.32)—Rump height	ThW (0.10)—Thorax width MaxOTL (0.09)—Max. oblique trunk length
III. Pelvis	—	PvL (0.33)—Pelvis length PvDI (0.24)—Pelvic dimension I	—
IV. Forelimbs	—	ScL (0.26)—Scapular length McC (0.21)—Metacarpal circumference ThUH (0.38)—Thoracic ungular height	McL (0.18)—Metacarpal length AmL (0.18)—Arm length
V. Hindlimbs	LgL (0.67)—Leg length	LgC (0.37)—Leg circumference MtL (0.23)—Metatarsal length	PvUH (0.13)—Pelvic ungular height

Standard errors for estimated heritability coefficients: StH: 0.32; WH: 0.31; LgL: 0.30; NVL: 0.30; ThD: 0.30; MWII: 0.29; ZW: 0.29; BH: 0.29; ThUH: 0.28; LgC: 0.28; HL: 0.28; PvL: 0.28; RH: 0.28; ScL: 0.27; PvDI: 0.27; MtL; 0.27; McC: 0.27; McL, AmL, PvUH, ThW: 0.26; MaxOTL, MDI, NFcD: 0.25.

## Data Availability

The data presented in this paper are available upon request from the corresponding author (E.P.).

## Supplementary Materials

The following supporting information can be downloaded at: https://www.mdpi.com/article/10.3390/ani16081190/s1, Table S1: Pedigree and demographic data of Polish Konik horses (ID 1-172).

## Abbreviations

The following abbreviations are used in this manuscript:

FAO	Food and Agriculture Organization
PCA	Principal Component Analysis
DA	Discriminant Analysis
PASc	Polish Academy of Sciences
RPN	Roztocze National Park
Wlkp	Wielkopolskie Voivodship
BH	Back height
MaxOTL	Maximal oblique trunk length
MinOTL	Minimal oblique trunk length
RH	Rump height
StH	Sternal height
ThD	Thorax depth
WH	Withers height
FamC	Forearm circumference
LgC	Leg circumference
LPvPMPh	Length of pelvic proximal and middle phalanx
LThPMPh	Length of thoracic proximal and middle phalanx
MDI	Mandibular dimension I
MDII	Mandibular dimension II
MWI	Mandibular width I
MWII	Mandibular width II
McC	Metacarpal circumference
McL	Metacarpal length
MtC	Metatarsal circumference
MtL	Metatarsal length
NFcD	Naso-facial dimension
NLL	Neck lateral length
NVL	Neck ventral length
PvAuL	Pelvic autopodium length
PvUH	Pelvic ungular height
ThAuL	Thoracic autopodium length
ThUH	Thoracic ungular height
ThC	Thorax circumference
AmL	Arm length
FcW	Facial width
FamL	Forearm length
HL	Head length
LgL	Leg length
PvDI	Pelvic dimension I
PvDII	Pelvic dimension II
PvL	Pelvis length
PvW	Pelvis width
ScL	Scapular length (with withers)
TL	Thigh length
ThW	Thorax width
ZW	Zygomatic width
*Ne*	Effective population size
REML	Restricted maximum likelihood
DMU	Derivative-free Mixed Unit
AI	Average information
SAS	Statistical software package
*n*	Number of horses
SD	Standard deviation
∑	Overall mean
F	F-value
*p*	*p*-level
Df	Degrees of freedom
s	Sieraków
p	Popielno
d	Dobrzyniewo
k	Kobylniki
*h*^2^	Heritability coefficients
EU	European Union
