# Supplementary material for: Morphometric Characterization and Preliminary Heritability Estimates of Body Measurements in the Polish Konik Populations
Source: Animals (Basel). 2026 Apr 14;16(8):1190. doi: 10.3390/ani16081190 (PMC13114266; doi:10.3390/ani16081190)
Supplement: Supplementary file 1 [file animals-16-01190-s001.zip › animals-4037181-supplementary.pdf]

Table S1. Pedigree and demographic data of Polish Konik horses (ID 1-172).

LP	Age	Sex	Center	Female Line	Male Line	Family	Strain	Mother (out of)	Father (by)
1	5.5	mare	s	KAROLKA		PADWA	LUZAK	Podaga out of Mruk	Jermak by Moryc
2	4	mare	s	WOLA		WILGA	LUZAK	Wigra out of Hawr	Jermak by Moryc
3	5	mare	s	URSZULKA		HALNA	LUZAK	Huma out of Trucht	Jermak by Moryc
4	6	mare	s	ZAZA		MORTA	LUZAK	Monika out of Trucht	Jermak by Moryc
5	14.5	mare	s	ZAZA		MORTA	NIŻOWIEC	Motia out of Luzak	Trucht by Niżowiec
6	12.5	mare	s	ZAZA		MORTA	NIŻOWIEC	Moreska out of Juhas	Trucht by Niżowiec
7	9.5	mare	s	TARPANKA I		JAWAJKA	ODMĘT	Jena out of Nordo	Mruk by Odmęt
8	3	mare	s	TARPANKA I		JAWAJKA	LUZAK	Jara out of Mruk	Jermak by Moryc
9	10	mare	s	TARPANKA I		JAWAJKA	JASPIS	Jerka out of Trucht	Hebel by Halicz
10	4.5	mare	s	KAROLKA		PADWA	NIŻOWIEC	Potia out of Kliper	Jenek by Trucht
11	3	mare	s	KAROLKA		PADWA	LUZAK	Patyna out of Hebel	Jermak by Moryc
12	3.5	mare	s	ZAZA		MORTA	JASPIS	Malina out of Trucht	Hebel by Halicz
13	7	mare	s	TARPANKA I		JAWAJKA	JASPIS	Jesień out of Kliper	Hebel by Halicz
14	9.5	mare	s	WOLA		WILGA	JASPIS	Widia out of Trucht	Hebel by Halicz
15	8.5	mare	s	KAROLKA		PADWA	JASPIS	Podaga out of Mruk	Hebel by Halicz
16	4.5	mare	s	KAROLKA		PADWA	LUZAK	Podaga out of Mruk	Jermak by Moryc
17	8	mare	s	KAROLKA		PADWA	NIŻOWIEC	Porada out of Moryc	Trucht by Niżowiec
18	8	mare	s	URSZULKA		HALNA	NIŻOWIEC	Horsa out of Gong	Trucht by Niżowiec
19	13	mare	s	WOLA		WILGA	NIŻOWIEC	Widawa out of Kliper	Trucht by Niżowiec
20	11.5	mare	s	ZAZA		MORTA	NIŻOWIEC	Moreska out of Juhas	Trucht by Niżowiec
21	12.5	mare	s	TARPANKA I		JAWAJKA	ODMĘT	Jerka out of Trucht	Mruk by Odmęt
22	6	mare	s	ZAZA		MORTA	JASPIS	Motawa out of Trucht	Hebel by Halicz
23	4	mare	s	ZAZA		MORTA	JASPIS	Malina out of Trucht	Hebel by Halicz
24	13.5	mare	s	KAROLKA		PADWA	ODMĘT	Podwika out of Nadir	Mruk by Odmęt
25	4	mare	s	ZAZA		MORTA	JASPIS	Motawa out of Trucht	Hebel by Halicz
26	7	mare	s	WOLA		WILGA	LUZAK	Wigra out of Hawr	Jermak by Moryc
27	9.5	stallion	s		WICEK	ODA	ODMĘT	Parna out of Trębacz	Mikrus by Kołczan
28	3.5	stallion	s		WICEK	MORTA	JASPIS	Monika out of Trucht	Hebel by Halicz
29	7.5	stallion	s		MYSZAK	JAWAJKA	LUZAK	Jara out of Mruk	Jermak by Moryc
30	13.5	stallion	s		WICEK	HALNA	ODMĘT	Horsa out of Gong	Mruk by Odmęt
31	6	mare	rpn	ZAZA		HOŁOBLA	ODMĘT	Hańcza out of Test ex Mech	Hektor by Trzmiel
32	4	mare	rpn	TRASZKA		TURZYCA	MORS I	Trzmielina out of Mohacz	Hart by Kordon
33	4	mare	rpn	ZAZA		HOŁOBLA	MORS I	Hańcza out of Test ex Mech	Hart by Kordon
34	4	mare	rpn	URSZULKA		HALNA	MORS I	Hurtnica out of Nochal	Hart by Kordon
35	6	mare	rpn	TARPANKA I		NIWA	ODMĘT	Natka out of Trzmiel	Homer ex Kolczak by Koleb.
36	8	mare	rpn	TRASZKA		TURZYCA	ODMĘT	Trzmielina out of Mohacz	Homer ex Kolczak by Koleb.
37	3	mare	rpn	TRASZKA		TURZYCA	ODMĘT	Toraczka out of Homer ex K.	Hektor by Trzmiel
38	8	mare	rpn	ZAZA		MORUSKA	ODMĘT	Morena out of Test ex Mech	Homer ex Kolczak by Koleb.
39	4	mare	rpn	TRASZKA		TURZYCA	ODMĘT	Toksja out of Homer ex K.	Hektor by Trzmiel
40	12	stallion	rpn		WICEK	HALNA	ODMĘT	Hubka out of Moloch	Trzmiel by Moloch
41	9.5	stallion	rpn		CHOCHLIK	HOŁOBLA	LAMUS	Hańcza out of Test ex Mech	Pałasz by Lamus
42	3.5	stallion	rpn		WICEK	TURZYCA	ODMĘT	Toksja out of Homer ex K.	Hektor by Trzmiel
43	6.5	gelding	rpn		WICEK	TURZYCA	ODMĘT	Trzmielina out of Mohacz	Hektor by Trzmiel
44	4	gelding	rpn		WICEK	NIWA	ODMĘT	Natka out of Trzmiel	Hektor by Trzmiel
45	4	gelding	rpn		GLEJT I	MORUSKA	MORS I	Mszyca out of Homer ex Kol.	Hart by Kordon

46	17	mare	p	BONA		BIBA	ODMĘT	Bierka out of Hultaj	Turkus by Odmęt
47	9	mare	p	TUNGUSKA		TUNDRA	LIŚCIAK	Tapioka out of Jaszcz	Homer by Tasznik
48	7	mare	p	POPIELICA		OPOSKA	LIŚCIAK	Odma out of Order	Osman by Tasznik
49	5	mare	p	TUNGUSKA		TUNDRA	LIŚCIAK	Tapioka out of Jaszcz	Homer by Tasznik
50	15	mare	p	DZINA I		DZISNA	PAJĄK	Dzisna out of Mors	Order by Tymian
51	16	mare	p	BIAŁKA		BUKWA	MORS I	Bukwa out of Komar	Turyn by Mors I
52	9	mare	p	MISIA II		LULA	LAMUS	Lotka out of Order	Tamir by Pałasz
53	4	mare	p	MISIA II		LULA	LIŚCIAK	Lotnica out of Tamir	Homer by Tasznik
54	12	mare	p	POPIELICA		OPOSKA	PAJĄK	Olcha out of Pałasz	Order by Tymian
55	4	mare	p	KAROLKA		JENA	BAJKO	Jętka out of Osman	Syriusz by Fornal
56	9	mare	p	KAROLKA		JENA	LIŚCIAK	Jejmościanka out of Lamus	Osman by Tasznik
57	8	mare	p	MISIA II		LULA	BAJKO	Lotka out of Order	Syriusz by Fornal
58	10	mare	p	KAROLKA		PADWA	BAJKO	Pochodnia out of Tulipan	Syriusz by Fornal
59	17	mare	p	MISIA II		LULA	PAJĄK	Lewkonia out of Nalewajko	Order by Tymian
60	8	mare	p	BIAŁKA		BORÓWKA	LIŚCIAK	Borówka out of Notabl	Osman by Tasznik
61	6	mare	p	DZINA I		DZISNA	BAJKO	Domena out of Okrzemek	Syriusz by Fornal
62	3	mare	p	POPIELICA		OPOSKA	LIŚCIAK	Odma out of Order	Homer by Tasznik
63	3	mare	p	DZINA I		DZISNA	BAJKO	Domena out of Okrzemek	Drzewiak by Syriusz
64	17	mare	p	GENEZA		GAZA	ODMĘT	Gazela out of Nordo	Hultaj by Odmęt
65	3.5	mare	p	GENEZA		GAZA	BAJKO	Groza out of Hultaj	Drzewiak by Syriusz
66	3.5	mare	p	TUNGUSKA		TUNDRA	LIŚCIAK	Tukana out of Syriusz	Homer by Tasznik
67	3.5	mare	p	BIAŁKA		BORÓWKA	BAJKO	Biruta out of Osman	Drzewiak by Syriusz
68	20	gelding	p		WICEK	JENA	NIŻOWIEC	Jedyna out of Jaspis	Niżowiec by Lazur
69	3.5	stallion	p		CHOCHLIK	NAGIETKA	LAMUS	Niecka out of Tulipan	Mor by Pałasz
70	6	stallion	p		MYSZAK	BORÓWKA	PAJĄK	Borówka out of Notabl	Lord by Order
71	3	stallion	p		CHOCHLIK	OPOSKA	LAMUS	Olza out of Osman	Trap by Pałasz
72	5	stallion	p		GLEJT I	KOKOSZKA	MORS I	Karina out of Hultaj	Baca by Narcyz
73	3	stallion	p		WICEK	BIBA	LIŚCIAK	Bielinka out of Turkus	Homer by Tasznik
74	11	stallion	p		LILIPUT	DZISNA	BAJKO	Drzewica out of Order	Syriusz by Fornal
75	9	stallion	p		WICEK	HALNA	LIŚCIAK	Hala out of Test	Tasznik by Liściak
76	24	gelding	p		WICEK	LESZCZYNA	NIŻOWIEC	Ożyna out of Oszczep	Tulipan by Jemiołuch
77	4	gelding	p		WICEK	OPOSKA	LIŚCIAK	Odma out of Order	Homer by Tasznik
78	6	gelding	p		LILIPUT	LULA	BAJKO	Lotka out of Order	Syriusz by Fornal
79	8	gelding	p		LILIPUT	LALKA	BAJKO	Lisica out of Order	Syriusz by Fornal
80	10	gelding	p		GORAJ	TRAWA	NIW	Turówka out of Liściak	Nacios by Niw
81	3	gelding	p		WICEK	TRAWA	LIŚCIAK	Turówka out of Liściak	Osowiec by Liściak
82	4	gelding	p		WICEK	TARNINA	LIŚCIAK	Tercja out of Liściak	Osowiec by Liściak
83	5	gelding	p		WICEK	OPOSKA	LIŚCIAK	Odma out of Order	Homer by Tasznik
84	8	gelding	p		WICEK	OPOSKA	LIŚCIAK	Odma out of Order	Osman by Tasznik
85	3.5	mare	k	ZAZA		MORUSKA	NIŻOWIEC	Mantyla out of Gong	Nort by Trucht
86	4.5	mare	k	TRASZKA		TURZYCA	NIŻOWIEC	Takieta out of Hebel	Nort by Trucht
87	3.5	mare	k	KAROLKA		DROSI	GORDYJ	Daria out of Gong	Kneź by Trębacz
88	5.5	mare	k	ZAZA		MORUSKA	NIŻOWIEC	Monta out of Gong	Nort by Trucht
89	5	mare	k	WOLA		WILGA	LUZAK	Wiena out of Tamir	Jermak by Moryc
90	5.5	mare	k	ZAZA		MORTA	NIŻOWIEC	Mołnia out of Hebel	Nort by Trucht
91	4.5	mare	k	ZAZA		MORUSKA	NIŻOWIEC	Mantyla out of Gong	Nort by Trucht
92	4.5	mare	k	KAROLKA		DROSI	GORDYJ	Droga out of Gong	Kneź by Trębacz
93	4.5	mare	k	KAROLKA		NALEWKA	GORDYJ	Nastula out of Moduł	Kneź by Trębacz

94	7.5	mare	k	TRASZKA		TURZYCA	GORDYJ	Tawra out of Moduł	Kneź by Trębacz
95	6.5	mare	k	ZAZA		MORUSKA	NIŻOWIEC	Monta out of Gong	Nort by Trucht
96	6.5	mare	k	TARPANKA I		NARTA	GORDYJ	Nigeria out of Gong	Kneź by Trębacz
97	6.5	mare	k	ZAZA		MORTA	NIŻOWIEC	Mołdawia out of Hebel	Nort by Trucht
98	6.5	mare	k	ZAZA		MORUSKA	MORS I	Modesta out of Trębacz	Gong by Mohacz
99	3	mare	k	ZAZA		MORUSKA	NIW	Murena out of Trębacz	Knop by Nadziak
100	4.5	mare	k	TRASZKA		TURZYCA	NIW	Terma out of Kordiał	Knop by Nadziak
101	7.5	mare	k	TRASZKA		TURZYCA	MORS I	Tajga out of Nadziak	Gong by Mohacz
102	7.5	mare	k	LILIPUTKA I		KISZKA	NIW	Katoda out of Trębacz	Knop by Nadziak
103	8.5	mare	k	KAROLKA		NALEWKA	MORS I	Nastka out of Magis	Gong by Mohacz
104	6.5	mare	k	TRASZKA		TURZYCA	LIFOK	Takieta out of Hebel	Jemen by Kliper
105	8.5	mare	k	ZAZA		MORTA	JUHAS	Meduza out of Gil	Topaz by Juhas
106	8.5	mare	k	ZAZA		MORTA	JUHAS	Mołnia out of Hebel	Topaz by Juhas
107	8.5	mare	k	TARPANKA I		NARTA	GORDYJ	Nigeria out of Gong	Kneź by Trębacz
108	7.5	mare	k	URSZULKA		HALNA	MORS I	Hajfa out of Nadziak	Gong by Mohacz
109	8.5	mare	k	KAROLKA		DROSI	MORS I	Daga out of Nadziak	Gong by Mohacz
110	8.5	mare	k	LILIPUTKA I		KISZKA	NIW	Katoda out of Trębacz	Knop by Nadziak
111	9.5	mare	k	TRASZKA		TURZYCA	NIŻOWIEC	Tawerna out of Narcyz	Kordiał by Trucht
112	9.5	mare	k	ZAZA		MORUSKA	JUHAS	Modelka out of Odmęt	Topaz by Juhas
113	9.5	mare	k	URSZULKA		HALNA	MORS I	Hajfa out of Nadziak	Gong by Mohacz
114	10.5	mare	k	KAROLKA		DROSI	MORS I	Daga out of Nadziak	Gong by Mohacz
115	9.5	mare	k	LILIPUTKA I		KISZKA	NIŻOWIEC	Kamea out of Halicz	Kordiał by Trucht
116	10.5	mare	k	LILIPUTKA I		KISZKA	MORS I	Klamra out of Halicz	Gong by Mohacz
117	10.5	mare	k	ZAZA		MORUSKA	MORS I	Montana out of Odmęt	Gong by Mohacz
118	10.5	mare	k	KAROLKA		NALEWKA	NIŻOWIEC	Nastka out of Magis	Moduł by Trucht
119	11.5	mare	k	TARPANKA I		NARTA	MORS I	Niobe out of Odmęt	Gong by Mohacz
120	12.5	mare	k	ZAZA		MORUSKA	GORDYJ	Mutra out of Huron	Trębacz by Tulipan
121	3.5	mare	k	KAROLKA		DROSI	GORDYJ	Droga out of Gong	Kneź by Trębacz
122	5.5	stallion	k		CHOCHLIK	NALEWKA	LIFOK	Newa out of Nadziak	Jemen by Kliper
123	10	stallion	k		LILIPUT	OPOSKA	BAJKO	Obrona out of Niżowiec	Syriusz by Fornal
124	3.5	stallion	k		GORAJ	NARTA	GORDYJ	Nigeria out of Gong	Kneź by Trębacz
125	4.5	stallion	k		WICEK	MORTA	NIŻOWIEC	Mołdawia out of Hebel	Nort by Trucht
126	6.5	stallion	k		CHOCHLIK	MORUSKA	LIFOK	Murena out of Trębacz	Jemen by Kliper
127	3	gelding	k		WICEK	NARTA	NIŻOWIEC	Niwea out of Gong	Nort by Trucht
128	3	gelding	k		WICEK	MORUSKA	NIŻOWIEC	Manita out of Gong	Nort by Trucht
129	3	gelding	k		GORAJ	KISZKA	NIW	Kasta out of Trębacz	Knop by Nadziak
130	3	gelding	k		WICEK	TURZYCA	NIŻOWIEC	Takieta out of Hebel	Nort by Trucht
131	3	gelding	k		WICEK	MORUSKA	NIŻOWIEC	Monta out of Gong	Nort by Trucht
132	3	gelding	k		CHOCHLIK	NALEWKA	JUHAS	Newa out of Nadziak	Mobil by Topaz
133	4.5	mare	d	TYGRYSKA		GRUSZA	NIW	Greja out of Nobis	Jehol by Nadziak
134	9.5	mare	d	TYGRYSKA		GRUSZA	BAJKO	Gryka out of Kołczan	Nobis by Syriusz
135	5.5	mare	d	TYGRYSKA		GRUSZA	NIW	Gawra out of Jaszczyk	Jehol by Nadziak
136	13.5	mare	d	TYGRYSKA		GRUSZA	NIW	Gryka out of Kołczan	Jehol by Nadziak
137	9.5	mare	d	TYGRYSKA		GRUSZA	BAJKO	Gayga out of Kołczan	Nobis by Syriusz
138	6.5	mare	d	LILIPUTKA I		KISZKA	BAJKO	Klaksa out of Mikrus	Nobis by Syriusz
139	7.5	mare	d	URSZULKA		HALNA	BAJKO	Hawra out of Trębacz	Nobis by Syriusz
140	7.5	mare	d	LILIPUTKA I		KISZKA	ODMĘT	Klika out of Jehol	Mikrus by Kołczan
141	9.5	mare	d	TARPANKA I		ODA	BAJKO	Pampa out of Jehol	Nobis by Syriusz

142	10.5	mare	d	TUNGUSKA		TUNDRA	ODMĘT	Tara out of Moryc	Mikrus by Kołczan
143	7.5	mare	d	TARPANKA I		ODA	NIW	Panela out of Mikrus	Jehol by Nadziak
144	4.5	mare	d	TYGRYSKA		GRUSZA	LIŚCIAK	Gruzinka out of Nobis	Komes by Jaszczyk
145	3.5	mare	d	TUNGUSKA		TUNDRA	NIW	Talia out of Jaszczyk	Jehol by Nadziak
146	3.5	mare	d	URSZULKA		HALNA	LIŚCIAK	Hazena out of Nobis	Komes by Jaszczyk
147	3.5	mare	d	KAROLKA		NALEWKA	BAJKO	Namiastka out of Mikrus	Noktus by Nobis
148	6.5	mare	d	TUNGUSKA		TUNDRA	NIW	Tera out of Mikrus	Jehol by Nadziak
149	5.5	mare	d	TARPANKA I		NUTA	BAJKO	Nilka out of Mikrus	Noktus by Nobis
150	11.5	mare	d	TARPANKA I		NUTA	ODMĘT	Nitka out of Mohacz	Mikrus by Kołczan
151	8.5	mare	d	KAROLKA		NALEWKA	BAJKO	Nokia out of Jehol	Nobis by Syriusz
152	4.5	mare	d	KAROLKA		NALEWKA	ODMĘT	Norika out of Nobis	Parwus by Mikrus
153	6.5	mare	d	TARPANKA I		ODA	BAJKO	Panela out of Mikrus	Nobis by Syriusz
154	13.5	mare	d	URSZULKA		HALNA	NIW	Hasta out of Mohacz	Jehol by Nadziak
155	5.5	mare	d	TARPANKA I		ODA	NIW	Panela out of Mikrus	Jehol by Nadziak
156	3.5	mare	d	TUNGUSKA		TUNDRA	LIŚCIAK	Turcja out of Jehol	Komes by Jaszczyk
157	3.5	mare	d	TYGRYSKA		GRUSZA	NIW	Galia out of Nobis	Jehol by Nadziak
158	3.5	mare	d	TARPANKA I		ODA	ODMĘT	Panama out of Nobis	Parwus by Mikrus
159	3	mare	d	KAROLKA		PADWA	LUZAK	Patyna out of Hebel	Jermak by Moryc
160	9.5	mare	d	TUNGUSKA		TUNDRA	NIW	Tamiza out of Mikrus	Jehol by Nadziak
161	5.5	mare	d	KAROLKA		NALEWKA	ODMĘT	Nokia out of Jehol	Mikrus by Kołczan
162	11.5	mare	d	TUNGUSKA		TUNDRA	LIŚCIAK	Tamiza out of Mikrus	Jaszczyk by Tasznik
163	4.5	mare	d	TUNGUSKA		TUNDRA	LIŚCIAK	Turcja out of Jehol	Komes by Jaszczyk
164	11.5	mare	d	KAROLKA		NALEWKA	LIŚCIAK	Namiastka out of Mikrus	Jaszczyk by Tasznik
165	10.5	stallion	d		WICEK	KISZKA	LIŚCIAK	Klaksa out of Mikrus	Jaszczyk by Tasznik
166	7.5	stallion	d		LILIPUT	NUTA	BAJKO	Nilka out of Mikrus	Nobis by Syriusz
167	6.5	stallion	d		CHOCHLIK	MORUSKA	LIFOK	Mantyla out of Gong	Jemen by Kliper
168	5.5	stallion	d		WICEK	MORTA	JASPIS	Motawa out of Trucht	Hebel by Halicz
169	3.5	stallion	d		LILIPUT	KISZKA	BAJKO	Klipa out of Mikrus	Noktus by Nobis
170	11	stallion	d		GORAJ	NALEWKA	NIW	Nawa out of Lifok	Jehol by Nadziak
171	10	gelding	d		WICEK	ODA	ODMĘT	Parna out of Trębacz	Mikrus by Kołczan
172	4.5	stallion	d		GORAJ	KISZKA	NIW	Kulka out of Mikrus	Jehol by Nadziak
